# Supplementary material for: An electrocardiography score predicts heart failure hospitalization or death beyond that of cardiovascular magnetic resonance imaging
Source: Sci Rep. 2022 Nov 1;12:18364. doi: 10.1038/s41598-022-22501-9 (PMC9626618; doi:10.1038/s41598-022-22501-9)
Supplement: Supplementary file 1 — Supplementary Information. [file 41598_2022_22501_MOESM1_ESM.docx]

**Supplemental material**

**Detailed description of CMR methodology**

CMR images were acquired using a 1.5 Tesla scanner (Magnetom Espree, Siemens Healthcare, Erlangen, Germany) and a 32-channel phased array cardiovascular coil. Examinations included standard breath held segmented cine imaging with steady-state free precession (SSFP) (1). LV mass, volumes and ejection fraction were measured from short-axis stacks of end-systolic and end-diastolic cine frames. LGE imaging was performed ten minutes after a 0.2 mmol/kg intravenous gadoteridol bolus (Prohance, Bracco Diagnostics, Princeton, NJ, USA), with a phase-sensitive inversion recovery pulse sequence to optimize LGE by rendering signal intensity proportional to T1 recovery. When patients could not breath hold, single-shot SSFP, and averaged phase-sensitive inversion recovery, motion corrected images were acquired (2). Typical acquisition parameters have previously been described (1).

*Quantification of the myocardial extracellular volume fraction*

An ECG-gated single-shot-modified Look Locker inversion recovery sequence (MOLLI) was used to acquire quantitative T1 maps. A native T1 map was acquired followed by a post-contrast T1 map after a gadolinium bolus injection. ECV was calculated in myocardium by delineating areas without LGE and calculated as: $ECV=\lambda\cdot(1-hematocrit)$, where λ = ΔR1myocardium/ΔR1bloodpool and ΔR1=1/T1_postcontrast_ - 1/T1_precontrast_ (3). Normal ECV was defined as <28.5% based on local scanning of healthy volunteers on the same scanner. Myocardial infarction and non-ischemic scar were defined as areas with LGE, and the extent of LGE was assessed by visual semi-quantitative estimation as previously described (4), and the middle third of the myocardium was traced for ECV measurements to avoid partial volume effects (5).

For blood ECV calculations, a circular region was traced in the middle of the blood pool to avoid partial volume effects by papillary muscles. The final ECV values were averaged from the basal and mid-ventricular short axis slices. Hematocrit measures were acquired on the day of CMR scanning. CMR data were analyzed using a commercial workstation (Leonardo, Siemens Healthcare, Erlangen, Germany).

*Quantification of global longitudinal strain*

GLS analysis was performed using semi-automated tissue feature tracking software (CVi42, Circle Cardiovascular Imaging Inc., Calgary, Canada). Epicardial and endocardial borders in the end-diastolic phase were manually traced in 2-, 3-, and 4-chamber views. Strain analyses tracings were inspected visually throughout the cardiac cycle, and manual changes were made when traces deviated from myocardial movement.

**Additional ECG score**

The 4-parametric ECG score for 1-year event had an AUC of 0.80 (0.72–0.87), and included 1) the frontal plane QRS-T angle (degrees), and 2) the heart rate corrected (Bazett) QT duration (ms), 3) the R wave duration in lead V2 (ms), and 4) the direction of the T loop in the left sagittal plane at its maximum voltage (degrees), and was calculated as:

Score = $\frac{1}{1+e^{-(Frontal QRS-T angle*0.011 + QTc*0.017 -8.1+R-duration+maximum T loop)}}*100$

**References Supplemental material**

1. Piehler KM, Wong TC, Puntil KS, Zareba KM, Lin K, Harris DM, et al. Free-breathing, motion-corrected late gadolinium enhancement is robust and extends risk stratification to vulnerable patients. Circ Cardiovasc Imaging. 2013;6(3):423-32.

2. Kellman P, Larson AC, Hsu LY, Chung YC, Simonetti OP, McVeigh ER, et al. Motion-corrected free-breathing delayed enhancement imaging of myocardial infarction. Magn Reson Med. 2005;53(1):194-200.

3. Arheden H, Saeed M, Higgins CB, Gao DW, Bremerich J, Wyttenbach R, et al. Measurement of the distribution volume of gadopentetate dimeglumine at echo-planar MR imaging to quantify myocardial infarction: comparison with 99mTc-DTPA autoradiography in rats. Radiology. 1999;211(3):698-708.

4. Schelbert EB, Piehler KM, Zareba KM, Moon JC, Ugander M, Messroghli DR, et al. Myocardial Fibrosis Quantified by Extracellular Volume Is Associated With Subsequent Hospitalization for Heart Failure, Death, or Both Across the Spectrum of Ejection Fraction and Heart Failure Stage. J Am Heart Assoc. 2015;4(12).

5. Fröjdh F, Fridman Y, Bering P, Sayeed A, Maanja M, Niklasson L, et al. Extracellular Volume and Global Longitudinal Strain Both Associate With Outcomes But Correlate Minimally. JACC Cardiovasc Imaging. 2020;13(11):2343-54.
